# Evaluating the Impact of Clonidine and Midazolam Premedication on Emergence Delirium in Pediatric Patients

**DOI:** 10.1002/pan.70275

**Published:** 2026-08-01

**Authors:** Anne Louise de Barros Garioud, Anwen Taplin, Yufei Shang, R. Nazim Khan, David Sommerfield, Neil Hauser, Aine Sommerfield, Britta S. von Ungern‐Sternberg

**Affiliations:** ^1^ Department of Anesthesiology, Juliane Marie Centre Copenhagen University Hospital—Rigshospitalet Copenhagen Denmark; ^2^ Department of Clinical Medicine University of Copenhagen Copenhagen Denmark; ^3^ Department of Anaesthesia and Pain Medicine Perth Children's Hospital, Child and Adolescent Health Service Perth Western Australia Australia; ^4^ Perioperative Medicine Team, The Kids Research Institute Australia Perth Western Australia Australia; ^5^ Institute for Paediatric Perioperative Excellence The University of Western Australia Perth Western Australia Australia; ^6^ School of Physics, Mathematics and Computing The University of Western Australia Perth Western Australia Australia; ^7^ Department of Mathematics and Statistics The University of Western Australia Perth Western Australia Australia; ^8^ Division of Emergency Medicine, Anaesthesia and Pain Medicine, Medical School The University of Western Australia Perth Western Australia Australia

**Keywords:** anesthesia, children, clonidine, emergence delirium, midazolam, premedication

## Abstract

**Background:**

Preoperative anxiety is common in children and is associated with emergence delirium—a distressing postoperative complication characterized by agitation and disorientation. Midazolam has long been the drug of choice for preoperative anxiolysis. More recently, α_2_ adrenoceptor agonists, such as clonidine, have gained popularity for their sedative and analgesic properties. The effect of these agents on the incidence of emergence delirium remains uncertain. This study evaluated whether premedication with clonidine or midazolam is associated with emergence delirium in children undergoing general anesthesia.

**Methods:**

We performed a post hoc analysis of prospectively collected data pooled from 10 clinical studies, comprising data from 4796 general anesthetics in children (0–16 years). The studies were carried out at a tertiary pediatric hospital in Perth, Australia. We assessed the impact of premedication with clonidine or midazolam on the incidence of emergence delirium using validated scales.

**Results:**

In this cohort, with a mean age of 6.8 (range: 0.03–16.97), 331 children received clonidine and 731 received midazolam. Overall, the incidence of emergence delirium was 6%. Primary propensity score–matched analyses found no significant association between clonidine (OR 1.19, 95% CI 0.65–2.16) or midazolam (OR 1.23, 95% CI 0.79–1.92) and emergence delirium. In our secondary adjusted logistic regression models, midazolam was associated with increased odds of emergence delirium, whereas clonidine showed no statistically significant association overall.

**Conclusions:**

The overall incidence of emergence delirium was low. While clonidine showed no statistically significant association with emergence delirium, our secondary multivariate analysis suggests midazolam might be associated with an increased risk of emergence delirium.

## Abstract (highlights)

**What Is Already Known About the Topic:**

Preoperative anxiety is common in children and is associated with emergence delirium. While midazolam has long been the drug of choice for preoperative anxiolysis, more recently, α_2_ adrenoceptor agonists, such as clonidine, have gained popularity for their sedative and analgesic properties. The effect of these agents on the incidence of emergence delirium remains uncertain.

**What New Information This Study Adds:**

This study suggests a possible advantage of premedication with clonidine compared with midazolam for the prevention of emergence delirium. While our primary analysis did not show an association between either premedication agent and emergence delirium, our secondary analyses suggest midazolam might be associated with higher odds in some subgroups.

**Abbreviations**
ASA: American Society of Anesthesiologists
CAPD: Cornell assessment of pediatric delirium
CI: confidence interval
ED: emergence delirium
ENT: ear‐nose‐throat
MICE: multiple imputation by chained equations
mYPAS: modified Yale Preoperative Anxiety Scale
OR: odds ratio
PAED: Pediatric Anesthesia Emergence Delirium
PONV: postoperative nausea and vomiting
UMSS: University of Michigan Sedation Scale

## 1 Introduction

The perioperative period can be a difficult and traumatic time for both young children and their families. Preoperative anxiety affects 40%–60% of preschool‐aged children [1, 2] and is a common challenge for anesthesiologists. Preoperative anxiety and distress can lead to a difficult induction of anesthesia. Importantly, anxiety and distress during induction are associated with emergence delirium (ED) and postoperative maladaptive behaviors, which may persist for up to 6 months after the day of surgery [3, 4]. Emergence delirium is a transient but distressing postoperative condition in children, characterized by agitation, inconsolability, and altered awareness. It can pose risks such as self‐injury, delayed recovery, and increased healthcare resource utilization [5]. In addition to preoperative anxiety, risk factors for ED include preschool age, sevoflurane‐based anesthesia, prior history of ED, and ear‐nose‐throat (ENT) or ophthalmology procedures [5, 6].

In addition to non‐pharmacological measures, benzodiazepines, and more specifically midazolam, have long been the first‐choice premedication in anxious children [7]. Midazolam is very lipophilic at physiological pH and therefore has a fast onset. It has excellent anxiolytic and sedative properties, but there have been concerns regarding paradoxical reactions as well as increased postoperative sedation and adverse postoperative behavioral changes [8]. In more recent years, α_2_ adrenoceptor agonists, notably clonidine and to some extent dexmedetomidine, have gained popularity as a sedative alternative to midazolam [9, 10]. In addition to its sedative properties, clonidine is widely used in pediatric anesthesia as a preventative measure for ED, for its analgesic and opioid‐sparing properties, and to prevent postoperative nausea and vomiting (PONV) [11]. Although clonidine has known preventive effects against ED, its effect when administered preoperatively in comparison with midazolam has mostly been investigated in smaller clinical studies [12, 13, 14, 15]. This study is a post hoc analysis of prospectively collected clinical research data investigating whether clonidine or midazolam premedication impacts the incidence of ED in a large cohort of pediatric patients undergoing general anesthesia. We hypothesize that premedication with clonidine might lower the risk of ED, whereas premedication with midazolam might increase the risk.

## 2 Patients and Methods

### 2.1 Study Design and Cohort

This pooled dataset examined the prospectively collected research records of pediatric patients aged 0–16 years who underwent general anesthesia at Perth Children's Hospital (or its predecessor, Princess Margaret Hospital for Children), a specialist tertiary children's hospital in Perth, Australia, between 2009 and 2024, and who were enrolled in previously conducted clinical studies by our research team. Data were extracted for a total of 4796 general anesthetics from 10 different studies that collected ED information as part of their original protocol. Each procedure was treated as an independent observation, regardless of potential repeated procedures in the same patient.

All included studies were individually approved by the Child and Adolescent Health Service Human Research Ethics Committee and the Institutional Review Board, were registered prospectively in the Australian New Zealand Clinical Trials Registry (www.anzctr.org.au) or ClinicalTrials.gov, and were performed in compliance with applicable legislation and institutional guidelines. Written consent was obtained before enrolment, except for the observational study POSSUM (see Appendix A), for which a waiver of consent was granted. All study procedures were carried out in accordance with the Declaration of Helsinki. The 10 studies are described, along with their registration numbers and references where possible, in Appendix A. The present study was separately approved by the site's Research Governance (RGS0000007094) and by the Child and Adolescent Health Service Human Research Ethics Committee of Perth Children's Hospital, 15 Hospital Avenue, Nedlands, Western Australia 6009 (Chairperson: Dr. Ed O'Loughlin) on July 18, 2024. We have followed the STROBE reporting guidelines for cross‐sectional analyses in the present manuscript [16].

### 2.2 Exposure

The exposure of interest was the oral administration of anesthetic premedication with clonidine or midazolam. The exposure was defined as binary, that is, based on premedication yes/no for either clonidine or midazolam, without including information on dosing. Other premedications (e.g., ketamine) were recorded and included as covariates in the analysis, but their effect on ED was not investigated.

### 2.3 Outcome

The primary outcome was the incidence of ED as defined in the original studies. Most of the studies assessed ED based on validated tools such as the Pediatric Anesthesia Emergence Delirium (PAED) scale [17], Cornell assessment of pediatric delirium (CAPD) [18], and Watcha [19]. Where a total PAED score was recorded, ED was defined as PAED ≥ 10 at any time in PACU, corresponding to the original scale validation. Using the CAPD scale, a score > 8 after 10 min from UMSS ≥ 2 (University of Michigan Sedation Scale [20]) was defined as ED. On the Watcha scale, ED was defined as ≥ 3.

### 2.4 Covariates

Variables included in the statistical analyses were:

- Age at time of surgery
- Year of operation
- Trial the participant was enrolled in
- Sex
- ASA (American Society of Anesthesiologists) physical status
- Surgical specialty
- Assessment tools used for emergence delirium
- Ketamine as premedication (yes/no)
- Anesthetic factors:
  ○ Induction method (inhalational or intravenous)
  ○ Length of anesthesia in minutes
  ○ Maintenance method (gas or total intravenous anesthesia (TIVA))
  ○ Intraoperative opioid analgesia (yes/no)

Preoperative anxiety assessments with the modified Yale Preoperative Anxiety Scale (mYPAS) were available for 598 surgeries and were explored in a sub‐analysis.

### 2.5 Statistical Analysis

All analyses were performed in R (https://www.R‐project.org/).

#### 2.5.1 Primary Analysis—Propensity Score Matching

Propensity score matching was performed to balance the covariates above, including age, sex, surgical type, and anesthetic maintenance. Separate propensity score‐matching models were used for each exposure (clonidine or midazolam) to allow evaluation of each premedication independently, and the presence of the opposite premedication was included as a covariate. Missingness in exposure, outcome, and covariates was handled using multiple imputation by chained equations (MICE) with *m* = 100 imputations, prior to estimating propensity scores. Propensity scores were estimated with a logistic regression model including all variables suspected to be related to the outcome, regardless of statistical significance [21]. The logit of estimated propensity scores was matched between exposed and non‐exposed groups using a 1:1 nearest neighbor approach without replacement, using a caliper of 0.2 × standard deviation. Balance was assessed using standardized mean differences (greatest < 0.1), variance ratios (greatest < 2), and graphical summaries. Finally, logistic regression models with a single covariate for premedication were fitted to estimate odds ratios (ORs) for ED incidence in the matched dataset. Findings were compared in the matched complete case dataset and multiply imputed datasets with *m* = 5, *m* = 20, and *m* = 100 imputations.

#### 2.5.2 Secondary Analysis—Multivariable Logistic Regression Analysis

Data were analyzed using a multivariable logistic regression model. The above‐mentioned covariates were considered for initial inclusion in the models and various interaction terms were investigated based on plots of the data. The tool used to assess delirium was excluded from this model as it was perfectly collinear with trial. The model was then reduced using stepwise selection based on the Akaike information criterion, following which further reduction was conducted manually based on *p* values to include only statistically significant covariates.

## 3 Results

### 3.1 Cohort and Descriptive Analyses

**FIGURE 1:**
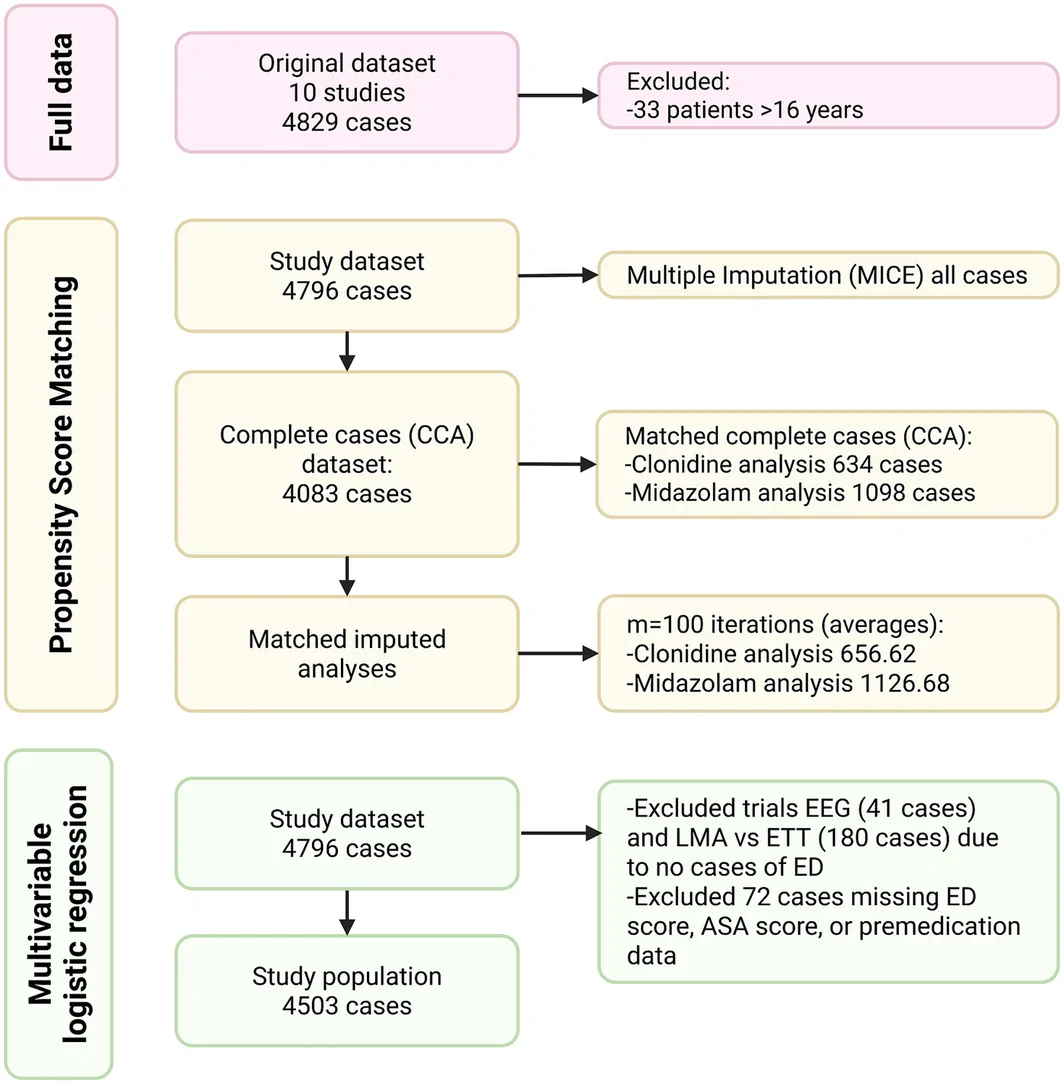
Datasets and study population. Illustration of the different datasets used for the different steps in propensity score matching analyses and to create the final study population for the multivariable logistic regression analysis.

Data on a total of 4796 general anesthetic exposures were collected. We found that 331 children received premedication with clonidine and 731 children received midazolam. Of these, 151 received both drugs preoperatively. The remaining 3881 received neither. Only four had missing data on premedication, and 77 had missing ED data. Table 1 describes baseline characteristics for the full cohort according to premedication status with either clonidine or midazolam. There were no cases of premedication with clonidine before 2019. Age distribution was comparable across groups, with the highest occurrence of premedication for either clonidine or midazolam in 0–6‐year‐olds (44% and 50% of clonidine and midazolam groups, respectively). Figure 1 further describes the different datasets and study population studied for each step of the analyses.

**TABLE 1:** Patient characteristics according to premedication received.

Characteristic	Overall	No clonidine	Clonidine	No midazolam	Midazolam
	*N* = 4796	*n* = 4461	*n* = 331	*n* = 4061	*n* = 731
Year of surgery					
2009–2013	627 (13%)	624 (14%)	0	623 (15%)	1 (0%)
2014–2017	688 (14%)	687 (15%)	0	534 (13%)	153 (21%)
2019–2024^a^	3481^b^ (73%)	3150 (71%)	331 (100%)	2904 (72%)	577 (79%)
Age	6.04 [3.36 to 9.98]	6.01 [3.28 to 10.02]	6.41 [4.16 to 9.38]	6.05 [3.07 to 10.24]	6.02 [4.06 to 8.63]
ASA^c^					
I–II	4164 (87%)	3895 (87%)	266 (80%)	3538 (87%)	623 (85%)
III–IV	625 (13%)	560 (13%)	65 (20%)	517 (13%)	108 (15%)
Sex, male	2775 (58%)	2580 (58%)	193 (58%)	2372 (58%)	401 (55%)
Inhalation induction	3653 (76%)	3375 (76%)	276 (83%)	3027 (75%)	624 (85%)
Sevoflurane maintenance	3637 (76%)	3406 (76%)	229 (69%)	3101 (76%)	534 (73%)
Anesthetic duration (min)	48 [30 to 76]	47 [30 to 75]	48 [32 to 82]	48 [30 to 75]	45 [29 to 78]
Surgical specialty					
ENT^d^	989 (21%)	922 (21%)	65 (20%)	832 (20%)	155 (21%)
General surgery/urology	776 (16%)	733 (16%)	41 (12%)	702 (17%)	72 (10%)
Dental	613 (13%)	533 (12%)	80 (24%)	446 (11%)	167 (23%)
Ophthalmologic	85 (2%)	85 (2%)	0	71 (2%)	14 (2%)
Other	2333 (49%)	2188 (49%)	145 (44%)	2010 (49%)	395 (44%)

*Note:* Data are *n* (%) and median [IQR]. The different shades are used to visually distinguish the overall cohort and the premedication comparison groups.

^a^ There were no data from 2018, 2020, and 2021.

^b^ One observational study contributed 2780 surgeries.

^c^ ASA: American Society of Anesthesiologists.

^d^ ENT: ear‐nose‐throat.

Emergence delirium was mostly assessed on validated scales: PAED (62%), CAPD (12%), Watcha (1%), and was retrieved from notes in patient files (as assessed on PAED) in 24% of cases. We found an overall incidence of emergence delirium of 6% (8% in children under 6 years), in line with previously recorded incidences at the institutions. The incidence did not vary by anesthesia maintenance method (6% for sevoflurane and TIVA maintenance). The occurrence of ED according to premedication status is described in Table 2 and according to trial and assessment tool in Appendix B.

**TABLE 2:** Emergence delirium incidence according to premedication received. Values are number (proportion).

Emergence delirium	Yes	No	Missing
Study dataset *N* = 4796	303 (6%)	4416 (92%)	77 (2%)
No premedication *N* = 2937	171 (6%)	2731 (93%)	35 (1%)
No clonidine *N* = 4461	272 (6%)	4118 (92%)	71 (2%)
Clonidine *N* = 331	31 (9%)	296 (89%)	4 (1%)
No midazolam *N* = 4061	193 (5%)	3813 (94%)	55 (1%)
Midazolam *N* = 731	110 (15%)	601 (82%)	20 (3%)
Clonidine and midazolam *N* = 151	17 (11%)	132 (87%)	2 (1%)
Missing *N* = 4	0	2 (50%)	2 (50%)

*Note:* Data are *n* (%).

### 3.2 Primary Analysis: Propensity Score Matching

#### 3.2.1 Premedication With Clonidine

Propensity score matching yielded a complete case dataset of 628 participants (314 who received clonidine and 314 who did not—but were as likely to have received according to the variables included in the model). After multiple imputation, the matched *m* = 100 dataset included an average of 655.64 cases with an overall ED incidence of 8.8%. All variables were balanced in the complete case analysis. However, in the *m* = 100 data, the variable for length of anesthesia was not balanced. Therefore, a multivariable model including a covariate for anesthesia duration was fitted for the final model of premedication against ED in the matched datasets.

The adjusted logistic regression analysis on the *m* = 100 dataset resulted in an odds ratio (OR) of 1.19 and 95% confidence interval (CI) of 0.65 to 2.16 and *p* value of 0.58 for the association between premedication with clonidine and ED. Logistic regression analyses on the complete case, *m* = 5, and *m* = 20 datasets yielded similar results.

#### 3.2.2 Premedication With Midazolam

The complete case dataset included 1106 participants, while the multiply imputed *m* = 100 dataset included an average of 1138.12, with an overall ED incidence of 8.9%. All variables were balanced in the complete case and multiply imputed datasets. The logistic regression analysis of the *m* = 100 matched data found an OR of 1.23 (95% CI 0.79 to 1.92, *p* value 0.36) for the association between premedication with midazolam and the incidence of ED in the *m* = 100 dataset, a finding that was confirmed similarly in the complete case, *m* = 5, and *m* = 20 datasets.

### 3.3 Secondary Analysis: Multivariable Logistic Regression

The associations between premedication and ED were further explored using multivariable logistic regression models. These analyses considered the full dataset with 4796 general anesthetic exposures to assess the association between premedication and the incidence of ED, adjusted for relevant confounders.

Two trials—EEG and LMA vs. ETT—had no observed cases of emergence delirium and therefore provided no variation in the outcome and did not contribute information to the logistic regression model. Therefore, surgeries from these two trials were excluded to stabilize model estimation and allow the remaining trial effects to be interpreted meaningfully. The sample size was thus reduced from 4796 to 4575 (95.4%), with 303 surgeries reported as having emergence delirium.

The logistic regression model for ED included age, premedication, surgical specialty, ASA, and trials. An interaction between premedication with clonidine and sex (Chi‐squared statistic = 5.44, df = 1, *p* = 0.02), and between premedication with midazolam and ASA (Chi‐squared statistic = 9.21, df = 2, *p* = 0.01) was also included in the model.

Premedication with clonidine was not statistically significantly associated with ED in males (OR 0.80, 95% CI 0.44 to 1.47, *p* = 0.48), but was related to a higher incidence of ED in females (OR 2.09, 95% CI 1.19 to 3.70, *p* = 0.01). A model without the interaction between sex and premedication with clonidine estimated no overall association between premedication with clonidine and ED (OR 1.26, 95% CI 0.82 to 1.91, *p* = 0.29). Premedication with midazolam appeared significantly associated with ED in patients with ASA I (OR = 1.85, 95% CI 1.20 to 2.85; *p* = 0.01), but was not significant in patients with ASA II (OR = 0.80, 95% CI 0.50 to 1.31, *p* = 0.38) or III/IV (OR = 1.96, 95% CI 0.93 to 4.16, *p* = 0.08). Age was inversely associated with decreasing odds of ED for increasing age groups, compared with 0–6‐year‐olds. Furthermore, we found that ENT surgery was statistically significantly associated with increased odds of ED compared to general surgery (OR 2.04, 95% CI 1.31 to 3.19), and gastroenterology surgery was statistically significantly associated with lower odds (OR 0.36, 95% CI 0.15 to 0.91), while all other surgical specialties had similar odds to general surgeries. Relative to the POSSUM trial, which was the trial with the largest sample size, most trials were associated with lower odds of emergence delirium, though this effect was not significant in MATES and VIVID. MIDAZ was the only trial that was associated with a higher odds of emergence delirium. The aim of the MIDAZ trial was to investigate a new pediatric formulation of midazolam, and all included participants were considered anxious preoperatively.

The results for the two propensity score‐matched models and the multivariable logistic regression model are presented in Table 3. An in‐depth description of statistical analyses and results is presented in Appendix C.

**TABLE 3:** Results for the different logistic regression models for association with emergence delirium.

Variable	Propensity score‐matched model (clonidine)		Propensity score‐matched model (midazolam)		Multivariable logistic regression model	
	OR (95% CI)	*p*	OR (95% CI)	*p*	OR (95% CI)	*p*
Clonidine premedication	1.19 (0.65 to 2.16)	0.580			Male: 0.80 (0.44 to 1.47) Female: 2.09 (1.19 to 3.70)	Male: 0.475 Female: 0.011
Midazolam premedication			1.23 (0.79 to 1.92)	0.356	ASA I: 1.85 (1.19 to 2.85) ASA II: 0.80 (0.50 to 1.31) ASA III or IV: 1.96 (0.93 to 4.16)	ASA I: 0.006 ASA II: 0.378 ASA III or IV: 0.079

*Note:* Data are odds ratio (95% confidence interval) and *p* values. The shading indicate cells where no estimate is applicable. In the clonidine propensity score‐matched model, midazolam use was balanced during propensity score matching and only the odds ratio for clonidine was estimated in the matched dataset. Therefore, no odds ratio is reported for midazolam. The same convention applies to the midazolam propensity score‐macthed model in which no odds ratio is reported for clonine.

### 3.4 Subgroup Analysis With Preoperative Anxiety Scores

A total of 598 participants were enrolled in studies, where preoperative anxiety was also recorded with a mYPAS score. The overall ED incidence in the subgroup was 17 (2.8%). Due to the low event rate, the relationship between premedication, anxiety scores, and ED could not be reliably analyzed in either the primary or secondary analyses.

## 4 Discussion

This post hoc analysis of a prospectively collected pooled data set explored the associations between clonidine and midazolam premedication and the incidence of emergence delirium.

With our primary analysis, we aimed to address potentially biased imbalances and missingness in the dataset with propensity score matching and multiple imputations by chained equations. In these analyses, we found no statistically significant association between either premedication and the incidence of ED (OR 1.23, 95% CI 0.79 to 1.92 for midazolam and OR 1.19, 95% CI 0.65 to 2.16 for clonidine). In our secondary analyses with a multivariable logistic regression model, we found that midazolam was statistically significantly associated with higher odds of ED (OR 1.85, 95% CI 1.20 to 2.85, *p* = 0.006), particularly in patients with ASA I. With this large dataset, we aimed to provide more solid data on the pros and cons of two of the most widely used pediatric premedication agents: clonidine and midazolam. While each has its own benefits, one does not appear definitively superior to the other according to the current literature. However, in terms of the prevention of ED, our data point towards a potential benefit of clonidine over midazolam—a finding that might be clinically important and deserves more investigation.

The search for the optimal premedication drug is far from concluded. A drug with strong anxiolytic properties, fast onset, ease of administration, high patient acceptability, good palatability if administered orally, and improved postoperative outcomes—including pain, nausea, and emergence behavior—would be a welcome addition in most pediatric anesthesiologists' toolboxes. The latest meta‐analysis directly comparing clonidine with benzodiazepines, including midazolam, favored clonidine in terms of sedation on induction, postoperative analgesia, and decreased emergence agitation [9]. Most clinical trials studying the effect on ED found that clonidine has a better or comparable effect [12, 13, 15], while one favored midazolam [14]. Some newer studies comparing dexmedetomidine with midazolam also reported lower [15, 22, 23, 24] incidences of emergence agitation with dexmedetomidine. Other studies comparing clonidine with midazolam highlight lower postoperative pain [25], less agitation on induction [26], but deeper sedation and less effective anxiolysis [27] with clonidine, and no difference in longer‐term behavioral outcomes [28]. More recent studies comparing the ultra‐short‐acting benzodiazepine remimazolam with dexmedetomidine [29] as premedication, found that both showed some promise for postoperative outcomes, with the possibility of intranasal administration. While this study assessed the potential postoperative effects of two of the most widely used premedications—midazolam and clonidine—we acknowledge that other agents like remimazolam and dexmedetomidine are gaining in popularity.

To analyze our observational dataset, in which the outcome data were not necessarily collected as primary outcomes, we used propensity score matching and a multivariable logistic regression model to adjust for baseline variables. Despite our efforts, due to the nature of the data, there may still be residual confounding at play. In particular, confounding by indication may have influenced the observed association between midazolam premedication and emergence delirium. Preoperative anxiety data were only available for a subset of the cohort, limiting our ability to fully account for this potential source of bias. Importantly, the included studies also differed in study design and setting and spanned a 15‐year period, during which pediatric anesthetic practice evolved substantially. Clonidine was first introduced in this dataset in 2019, increasing the risk of temporal confounding. Although we attempted to mitigate this through propensity score matching and adjustment for trial in the multivariable logistic regression, unmeasured and residual confounding may still have influenced the findings. We expect the primary analysis to better control for confounding, as the matched data contained a similar distribution of studies and year at surgery for participants with and without premedication. This could partly explain the more conservative estimates and results from the primary analysis compared to the secondary analysis. The smaller sample size in our matched data compared to the full complete case dataset would also contribute to the more conservative estimates in the primary analysis.

Furthermore, overall ED incidences were very low compared with commonly reported numbers of around 30% [6]. While this is somewhat surprising given the median age (6.04 years), the number of ENT cases (21% of all procedures), and the amount of sevoflurane maintenance (76%), the overall incidence is in line with previously reported incidences at these institutions. As a result of low event rates at our institution, the higher odds ratio found for premedication with midazolam equates to a small absolute risk difference (ED rate of 8% without premedication with midazolam and 9.7% with premedication in the *m* = 100 matched dataset), which may not translate to sites with a higher event rate. Thus, the low incidence of our primary outcome might have impacted the external validity of the study. The primary outcome was defined according to the scales employed in the included studies. In older studies, emergence delirium, assessed usually on the PAED scale, was retrieved from notes in the patients' files, which might have introduced a risk of misclassification. In addition, dosing data were not available in our analyses. Some studies point to a better effect of clonidine at 4 μg kg^−1^ compared with 2 μg kg^−1^ [12, 30]. It is institutional standard to administer 3–4 μg kg^−1^ clonidine and 0.5 mg kg^−1^ midazolam, but data were not systematically recorded in the original studies.

We acknowledge that this limits the granularity of our findings, where dose, timing, formulation, and route may have clinically important effects on emergence delirium risk. Finally, some children contributed more than one anesthetic exposure. Premedication administration varies between procedures, and the proportion of children having repeated exposures was low (11.8%). We therefore treated procedures as independent to prevent sample size loss. However, residual within‐patient correlation could have contributed to smaller standard errors in our results. Repeated measures modeling could have been used to further account for within‐patient correlation. However, the rarity of repeat presentations in our small dataset means the increased complexity of these models is unlikely to yield reliable estimates.

## 5 Conclusions

In conclusion, this study suggests a possible advantage of premedication with clonidine compared with midazolam for the prevention of emergence delirium. While our primary analysis did not show an association between either premedication agent and emergence delirium, our secondary analyses suggest midazolam might be associated with higher odds in some subgroups. It would be interesting to explore the comparative effects of premedication with α_2_‐adrenoceptor agonists and benzodiazepines, including newer drugs, in future large, randomized trials, including measurements of preoperative anxiety levels.

## Author Contributions

**Anne Louise de Barros Garioud:** conceptualization, methodology, data curation, writing – original draft, writing – review and editing, project administration. **Anwen Taplin:** methodology, validation, formal analysis, data curation, writing – original draft, writing – review and editing, visualization. **Yufei Shang:** methodology, validation, formal analysis, data curation, writing – review and editing, visualization. **R. Nazim Khan:** methodology, validation, writing – review and editing, supervision. **David Sommerfield:** conceptualization, methodology, writing – review and editing. **Neil Hauser:** writing – review and analysis. **Aine Sommerfield:** conceptualization, writing – review and editing. **Britta S. von Ungern‐Sternberg:** conceptualization, methodology, validation, resources, writing – review and editing, supervision, project administration, funding acquisition.

## Funding

We thank the following funders for their support of the included studies:

- Australian and New Zealand College of Anesthetists (ANZCA) Foundation: Normal Values and MIDAZ.
- Perth Children's Hospital Foundation (PCH Foundation), formerly known as the Princess Margaret Hospital Foundation (PMH Foundation): Magic Coat, EEG, and MATES.
- Society for Pediatric Anesthesia in New Zealand and Australia (SPANZA): EEG.
- The University of Western Australia (UWA) Pathfinder grant: MIDAZ.

ALBG research exchange to Perth Children's Hospital, Australia was funded by travel grants from: A.P. Møller Fonden for Lægevidenskabens Fremme, University of Copenhagen Graduate School, Reinholdt W. Jorck og Hustrus Fond, Det Lægevidenskabelige Fakultets Fond, Christian og Ottilia Brorsons Rejselegater, Familien Hede Nielsens Fond, and a Ph.D.‐stipend from Rigshospitalet Research Grants. BSvUS is partly funded by the Stan Perron Charitable Foundation and through a National Health and Medical Research Council Investigator Grant (Grant number: 2009322).

## Conflicts of Interest

BSvUS is a section editor for *Pediatric Anesthesia*.

## Figures and Tables

**FIGURE C.1:**
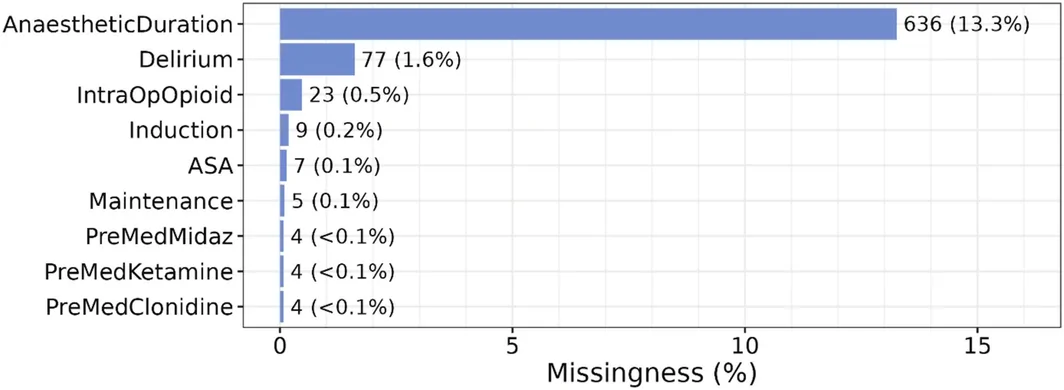
Percentage missing for each variable of the red panda dataset, the number of cases missing each variable is listed with the rounded percentage on the right of each bar. Only variables with at least one missing value are shown in the plot.

**FIGURE C.2:**
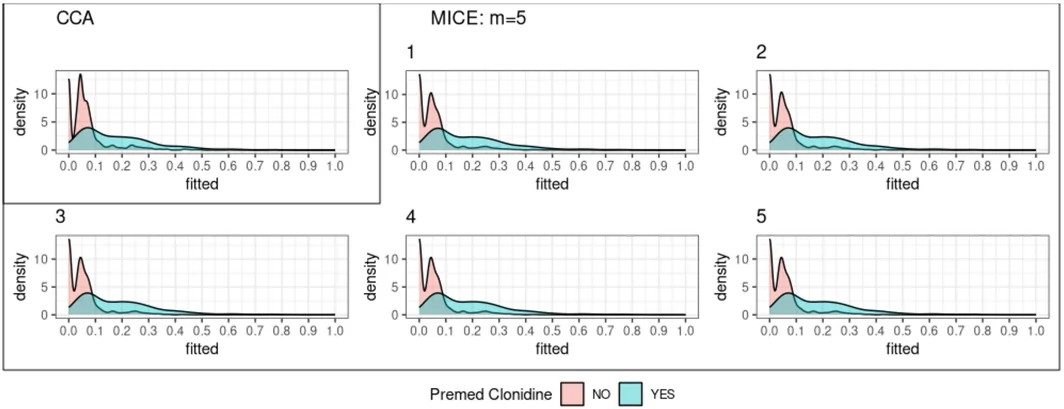
Density plots of the estimated propensity score of premedication with clonidine in the complete case and *m* = 5 imputed datasets.

**FIGURE C.3:**
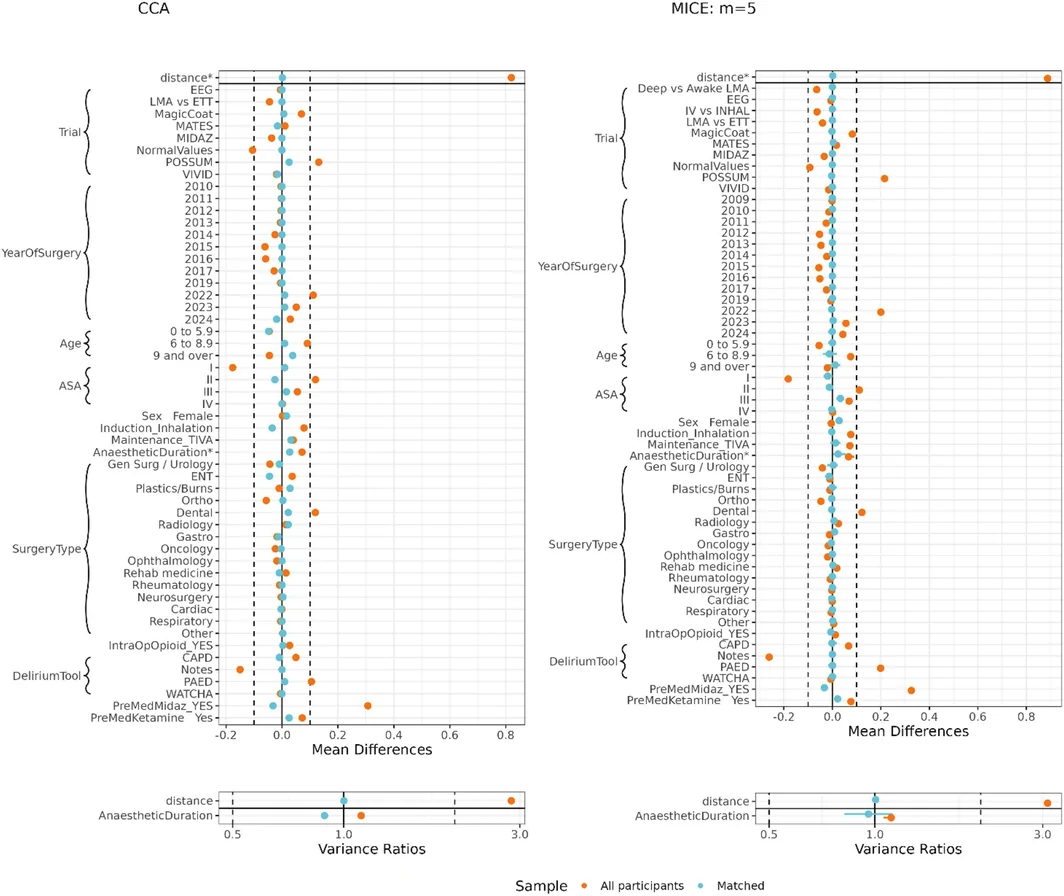
Covariate balance in the complete case and *m* = 5 imputation datasets after matching. The MICE *m* = 5 plot shows the range of mean differences in each of the five imputed datasets.

**FIGURE C.4:**
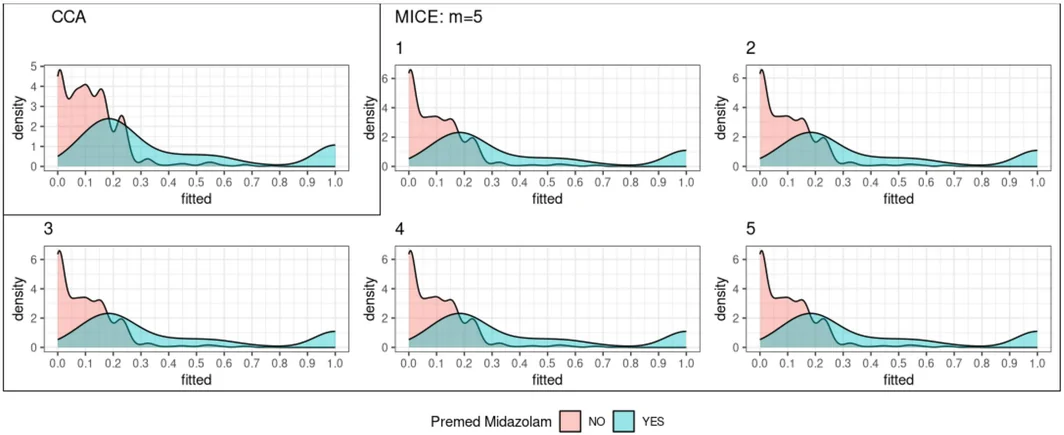
Density plots of the estimated propensity score of premedication with midazolam in the complete case and *m* = 5 imputed datasets.

**FIGURE C.5:**
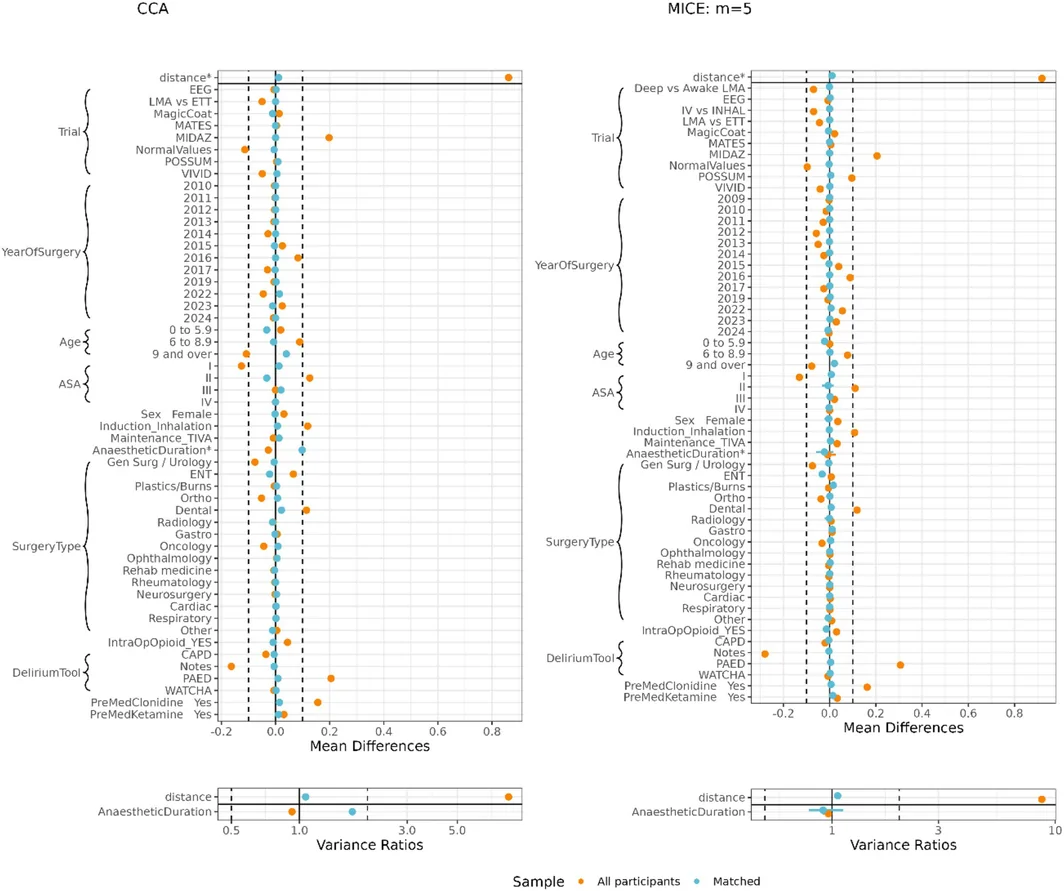
Covariate balance in the complete case and *m* = 5 imputation datasets after matching. The MICE *m* = 5 plot shows the range of mean differences in each of the five imputed datasets.

**FIGURE C.6:**
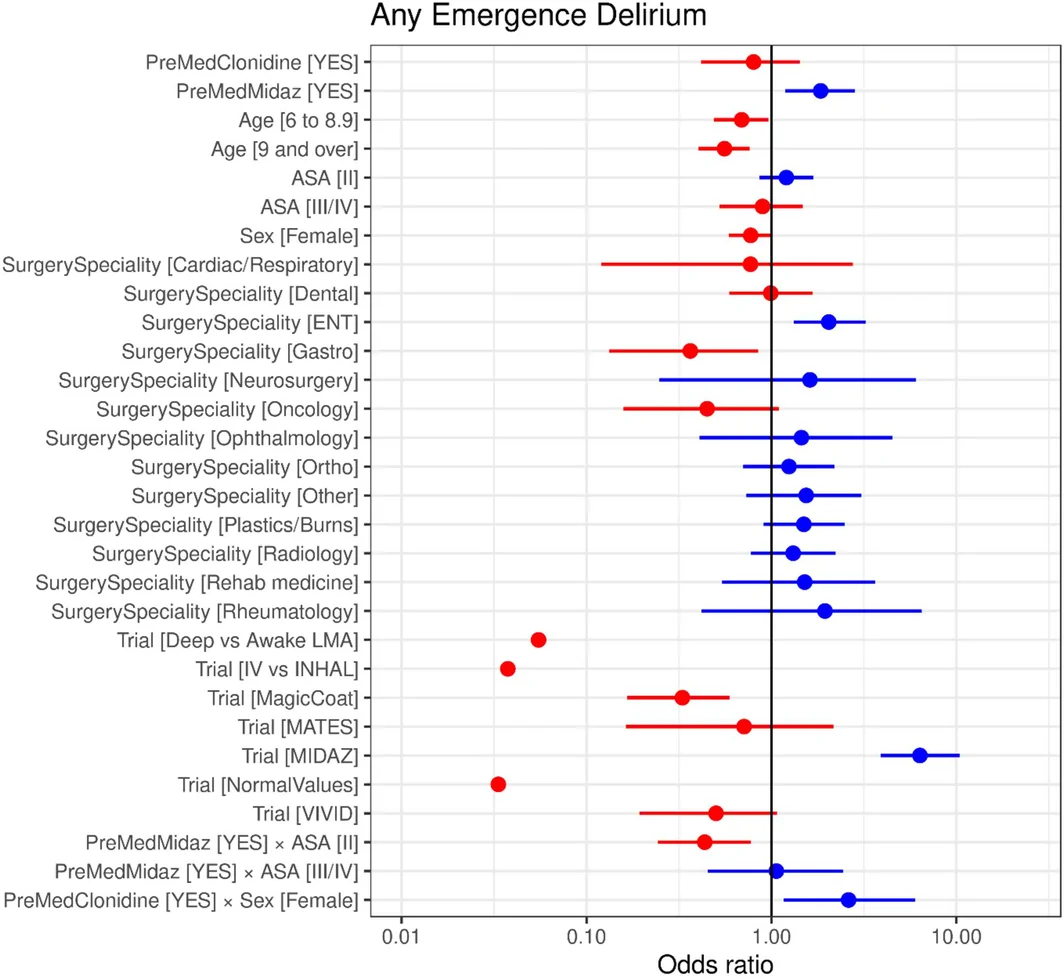
Forest plot of odds ratios from the logistic regression model to evaluate the association between covariates and ED. Each point represents an odds ratio with a 95% confidence interval. The confidence interval for some level within Trial is not shown due to its wide range.

## Data Availability

De‐identified participant data, including a data dictionary defining each field in the set, will be made available to others after publication of this article, upon reasonable request to the corresponding author at: britta.regli-vonungern@health.wa.gov.au and in line with institutional ethics and governance regulations. Data will be made available to researchers with a specified use approved by appropriate institutional review boards and with a signed data access agreement. The study protocol and statistical analysis plan will be made available on request.

## Appendix A Description of Clinical Studies Providing Data to the Pooled Dataset

**Table:**

Study acronym	Study registration	Study details	Publication
POSSUM	ACTRN12622000559718	Single‐center prospective observational cluster cohort study enrolling 3816 children aged 0–18 years of age undergoing elective and emergency surgery under general anesthesia in the operating theaters who will be transferred to PACU after surgery – between 8 am and 6 pm on weekdays.	In preparation
Magic Coat	ACTRN12623000403639	Sequential pre and post intervention prospective cohort clinical trial (*n* = 400) to examine the impact of an accessible, customizable online resource designed to introduce and build upon skills for communicating and managing anxiety among children in the perioperative period and improving induction compliance	In preparation
MATES	ACTRN12624000252516	Single‐center, double blinded, randomized, placebo‐controlled pilot trial on the effectiveness of melatonin to improve sleep quality and thereby decrease postoperative pain in 200 children aged 2–16 years following tonsillectomy.	Study ongoing
VIVID	ACTRN12622000085774	Single‐center, randomized, controlled, parallel‐group trial of 200 children aged 4–13 years randomly allocated to watch a 5‐min underwater‐themed video with breathing exercises during anesthetic induction on a Virtual Reality goggles or tablet device.	Samnakay S, von Ungern‐Sternberg BS, Evans D, Sommerfield AC, Hauser ND, Bell E, Khan RN, Sommerfield DL. 3‐Dimensional Virtual Reality Versus 2‐Dimensional Video for Distraction During the Induction of Anesthesia in Children to Reduce Anxiety: A Randomized Controlled Trial. *Anesthesia & Analgesia* 2024 Aug 23. https://doi.org/10.1213/ANE.0000000000007119
EEG	NCT03432351	Prospective observational cross‐sectional study	Yuan I, Xu T, Skowno J, Zhang B, Davidson A, von Ungern‐Sternberg BS, Sommerfield D, Zhang X, Zhang M, Zhao P, Oiu H, Jiang Y, Zuo Y, de Graaff JC, Vukskits L, Olbrecht VA, Szmuk P, Kurth C on behalf of BRAIN Collaborative Investigators. Isoelectric Electroencephalography in Infants and Toddlers During Anesthesia for Surgery: An International Observational Study. *Anesthesiology* 2022 Aug 1;137(2):187–200. https://doi.org/10.1097/ALN.0000000000004262
MIDAZ	ACTRN12615000225516	Prospective, open‐label, single‐center, randomized, single‐treatment trial of midazolam with 150 children assigned either as a chewable chocolate‐based tablet delivery system or midazolam solution.	Salman S, Tang EKY, Cheung LC, Nguyen MN, Sommerfield D, Slevin L, Lim LY, von Ungern‐Sternberg BS. Novel, Palatable Paediatric Oral Formulation of Midazolam: Pharmacokinetics, Tolerability, Efficacy and Safety. *Anaesthesia* 2018; 73: 1469–1477
IV vs. INHAL	ACTRN12610000252011	Single‐center open‐label randomized controlled trial (parallel groups) of 300 infants and children aged up to 8 years, with at least two parentally reported risk factors for perioperative respiratory adverse events and who were scheduled for elective surgery were eligible for recruitment.	Ramgolam A, Hall G, Zhang G, Hegarty M, Ramgolam A, Hall G, Zhang G, Hegarty M, von Ungern‐Stenberg BS. Inhalational Versus Intravenous Induction of Anaesthesia in Children With a High Risk of Perioperative Respiratory Adverse Events—A Randomized Controlled Trial. *Anesthesiology*. 2018 Jun;128(6):1065–1074. https://doi.org/10.1097/ALN.0000000000002152
LMA vs. ETT	ACTRN12610000250033	Single center, randomized controlled trial of 181 infants aged 0 to 12 months undergoing elective general anesthesia with or without regional or local anesthesia, assessed by their attending anaesthesiologist to be suitable to receive either supraglottic airway or an endotracheal tube, and if a fentanyl dose of 1 μg/kg or lower was expected to be needed.	Drake‐Brockman TFE, Ramgolam A, Zhang G, Hall GL, von Ungern‐Sternberg BS. The Effect of Endotracheal Tubes Versus Laryngeal Mask Airways on Perioperative Respiratory Adverse Events in Infants: A Randomised Controlled Trial. *The Lancet*. 2017 Feb 18;389(10070):701–708
DEEP vs. AWAKE LMA	ACTRN12609000387224	Single center open‐label parallel‐arm randomized controlled trial of 290 infants and children aged up to 16 years with at least one parentally reported risk factor for the perioperative respiratory adverse events and undergoing tonsillectomy with or without adenoidectomy and/or myringotomy.	Ramgolam A, Hall GL, Zhang G, Hegarty M, von Ungern‐Sternberg BS. Deep or Awake Removal of Laryngeal Mask Airways in Children at a High Risk of Respiratory Adverse Events Undergoing Tonsillectomy Procedures—A Randomised Controlled Trial. *British Journal of Anaesthesia*, 2018 Mar;120(3):571–580
Normal Values Study	ACTRN12614001252606	Single center observational study of 480 children at measuring lung function outcomes in healthy children aged between 1 and 15 years old who are undergoing surgery under general anesthesia and require mechanical ventilation.	In preparation

## Appendix B Emergence Delirium Incidence by Trial and Assessment Tool

**Table:**

	CAPD	PAED	Notes	WATCHA
Deep vs. Awake LMA	—	—	2/274 (0.7%)	—
EEG	—	—	—	0/40 (0.0%)
IV vs. INHAL	—	—	1/281 (0.4%)	—
LMA vs. ETT	—	—	0/168 (0.0%)	—
Magic coat	11/395 (2.8%)	—	—	—
MATES	—	3/25 (12.0%)	—	—
MIDAZ	—	53/139 (38.1%)	—	—
Normal values	—	—	1/414 (0.2%)	—
POSSUM	—	226/2794 (8.1%)	—	—
VIVID	6/189 (3.2%)	—	—	—
Total (column‐wise %)	17/584 (2.9%)	282/2958 (9.5%)	4/1137 (0.4%)	0/40 (0%)

*Note:* Data are proportion as *n*/*N* (%). Denominators exclude 77 patients with missing ED outcomes.

## Appendix C Statistical Methods, Analyses, and Results

### Data Processing

4829 surgeries were collated from the 10 studies. 33 patients from POSSUM were excluded as they were older than 16 and did not meet the inclusion criteria (see Appendix A for description of the included studies).

An additional four studies were previously used to fill in missing information for patients who were co‐enrolled in multiple studies. CHEETAH was used to fill in data for patients also enrolled in Magic Coat or MATES, while COMET, GIRAFFE, and NIGHTOWL were used to fill in data for patients also enrolled in POSSUM. Information on these studies can be found in Table C.1. Manuscripts for these four additional studies are currently in preparation.

**TABLE C.1:** Information on the studies used to provide additional information.

Study acronym	Study registration	Study details
CHEETAH	ACTRN12624000018516	Single‐center prospective observational cluster cohort study (*n* = 12 216) to establish the relationships between environmental exposures and risk of perioperative respiratory adverse events (PRAE) as well as socioeconomic factors and the risk of PRAE.
COMET	ACTRN12620000380998	Single‐center prospective observational study to determine whether it is feasible to continually track the changes in oscillatory mechanics of the respiratory system, as measured by continuous respiratory oscillometry in 60 children aged 2–16 years, undergoing laparoscopic appendectomy
GIRAFFE	ACTRN12622000471785	Pragmatic feasibility pilot study to assess whether total intravenous anesthesia (TIVA) and nasal high‐flow (NHF) are both feasible and non‐inferior compared to general anesthesia via a laryngeal mask airway (LMA) in 120 children presenting for an upper gastrointestinal endoscopy.
NIGHTOWL	ACTRN12620001190998	Single‐center, explorative, observational study of 60 children aged 1–8 years investigating the use of upper airway collapsibility, 3D facial photography, activity monitors, and nocturnal oximetry compared to polysomnography (PSG) to predict PRAE.

Each procedure was treated as an independent observation, regardless of potential repeated procedures in the same patient. We decided to include repeat presentations by participants in our analysis and ignore the correlations between these participants to prevent sample size loss (Table C.2).

**TABLE C.2:** The number of patients with multiple procedures within the dataset.

Number of procedures	Number of patients
1	4231
2	163
3	35
4	19
> 4	8

All variables were missing in less than 1% of cases, apart from Anesthetic Duration (length of anesthesia in minutes) which was missing in 13% of participants, and Delirium (emergence delirium as defined in Section 2.3 in the main manuscript), which was missing in 77 (1.7%) of cases. Anesthetic duration was mostly missing in the two older studies DEEP versus AWAKE LMA and IV versus INHAL, and these data could not be easily obtained as the surgeries occurred at Princess Margaret Hospital for Children, the predecessor to the current Perth Children's Hospital (Figure C.1).

### Primary Analysis—Propensity Score Matching

We performed propensity score matching (logistic regression to estimate propensity scores, 1:1 nearest neighbor matching within a 0.2 SD caliper, without replacement) in each imputed dataset [1]. Matching in older studies was limited by the very low rate of premedication with either midazolam or clonidine. In some of the older studies, no participants received premedication, which meant there were frequently no participants to match within the caliper distance used. Therefore, participants of these studies were rarely included in the matched dataset.

For both Clonidine and Midazolam, the propensity score models converged and achieved covariate balance. Multiple imputation by chained equations (MICE) using predictive mean matching was used to fill missing data in 15% of surgeries using the package MICE [2] in R. Variable selection for the MICE models followed a structured approach. We first considered which variables were relevant and required to be imputed or included in the MICE models as predictors. Predictors with a pairwise correlation magnitude greater than 0.2, along with predictors of those predictors, were initially included. When no predictors were found for a given variable, appropriate theoretical predictors were selected based on clinical relevance. The study outcome (Delirium) and Year of Surgery were included as predictors for all imputed variables. Predictors for each variable in the MICE imputations are listed in Table C.3.

**TABLE C.3:** Variables used in the predictive mean matching MICE.

Variable	Correlation > 0.2	MICE predictors
*ASA*	SurgerySpeciality	Surgery Speciality, Age, Induction, Maintenance, YearOfSurgery, Delirium
*PreMedClonidine*	PreMedMidaz	PreMedMidaz, Age, YearOfSurgery, Delirium
*PreMedMidaz*	PreMedClonidine	PreMedClonidine, Age, YearOfSurgery, Delirium
*PreMedKetamine*		PreMedClonidine, PreMedMidaz, Age, YearOfSurgery, Delirium
*Induction*	Age	Age, Maintenance, YearOfSurgery, Delirium
*Maintenance*	YearOfSurgery	Age, Induction, YearOfSurgery, Delirium
*IntraOpOpioid*		Age, Induction, Maintenance, SurgerySpeciality, YearOfSurgery, Delirium
*AnaestheticDuration*		Age, Induction, Maintenance, SurgerySpeciality, YearOfSurgery, Delirium
*Delirium*		Age, Induction, Maintenance, SurgerySpeciality, YearOfSurgery, Delirium

The packages MatchIt [3] and MatchThem [4] were used for propensity score matching in the complete case and multiply imputed datasets.

The complete cases analysis included 4083 of the 4796 surgeries (85%) where delirium scores should have been measured. The multiple imputation analyses used all 4796 surgeries.

#### Effect of Clonidine

Matching used a 1:1 ratio with a caliper of 0.2 × SD, and at least 98% of cases with Clonidine were matched across the complete case and multiply imputed datasets. A binomial logistic regression model was used to estimate propensity scores for the probability of pre‐medication with clonidine using the covariates *Age*, *YearOfSurgery*, *Sex*, *ASA*, *SurgerySpeciality*, *DeliriumTool* (the tool used to assess delirium), *PreMedKetamine* (yes/no), *Induction* (Inhalational/intravenous method), *AnaestheticDuration* (minutes), *Maintenance* (TIVA or gas method), *IntraopOpioid* (yes/no), *Trial*, *PreMedMidazolam* (yes/no).

All variables were balanced in the complete case and *m* = 5 and *m* = 20 imputations based on standardized mean difference and on variance ratio following matching. *AnaestheticDuration* was not balanced in the *m* = 100 imputation (standardized mean difference greater than 0.1). Therefore, multivariable model with a covariate for *AnaestheticDuration* was also fitted. Matching was performed separately within each multiply imputed dataset, and estimates were pooled using Rubin's rule and the *MatchThem* package across the *m* = 5, *m* = 20, and *m* = 100 imputations (Figures C.2 and C.3).

The number of matched patients in the complete case and multiple imputation analyses, as well as the pooled results of the final model against *Delirium* in each dataset, are shown in Table C.4. Using the matched complete case and imputed datasets, premedication with Clonidine was not associated with the incidence of emergence delirium.

**TABLE C.4:** Propensity score matched analysis results for premedication with Clonidine.

Dataset	Clonidine	Total, *n*	Matched, *n* (%)	Emergence delirium, *n* (%)	OR (95% CI)	*p*
CCA	No	3762.00	314 (8%)	27 (8.6%)		
	Yes	321.00	314 (98%)	31 (9.9%)	1.164 (0.68, 2.01)	0.582
*m* = 5	No	4464.80	327.6 (7%)	26 (7.9%)		
	Yes	331.20	327.6 (99%)	31 (9.5%)	1.22 (0.65, 2.27)	0.528
*m* = 20	No	4465.00	327.75 (7%)	25.6 (7.8%)		
	Yes	331.00	327.75 (99%)	30.9 (9.4%)	1.24 (0.65, 2.37)	0.514
*m* = 100	No	4464.97	327.82 (7%)	26.6 (8.1%)		
	Yes	331.03	327.82 (99%)	30.8 (9.4%)	1.19 (0.65, 2.16)^a^	0.580

^a^ Anesthesia duration was included as a covariate as it remained unbalanced in the matched multiple imputation datasets.

#### Effect of Midazolam

Matching used a 1:1 ratio with a caliper of 0.2 × SD, at least 78% of cases with Midazolam were matched across the complete case and multiply imputed datasets. A binomial logistic regression model was used to estimate propensity scores for the probability of premedication with Midazolam using the covariates *Age*, *YearOfSurgery*, *Sex*, *ASA*, *SurgerySpeciality*, *DeliriumTool* (the tool used to assess delirium), *PreMedKetamine* (yes/no), *Induction* (Inhalational/intravenous method), *AnaestheticDuration* (minutes), *Maintenance* (TIVA or gas method), *IntraopOpioid* (yes/no), *Trial*, *PreMedClonidine* (yes/no).

All variables were balanced in the complete case and imputed datasets based on standardized mean difference and variance ratio following matching (Figures C.4 and C.5).

The number of matched patients in the complete case and multiple imputation analyses, as well as the pooled results of the final model against *Delirium* in each dataset, are shown in Table C.5. Using the matched complete case and imputed datasets, premedication with midazolam was not associated with the incidence of emergence delirium.

**TABLE C.5:** Propensity score matched analysis results.

Dataset	Midazolam	Total, *n*	Matched, *n* (%)	Emergence delirium, *n* (%)	OR (95% CI)	*p*
CCA	No	3381.00	553 (16%)	43 (7.8%)		
	Yes	702.00	553 (79%)	55 (9.9%)	1.31 (0.86, 2)	0.205
*m* = 5	No	4064.80	569.4 (14%)	43.2 (7.6%)		
	Yes	731.20	569.4 (78%)	55.2 (9.7%)	1.31 (0.81, 2.12)	0.262
*m* = 20	No	4064.95	569 (14%)	46.5 (8.2%)		
	Yes	731.05	569 (78%)	55.1 (9.7%)	1.21 (0.78, 1.87)	0.390
*m* = 100	No	4064.97	569.06 (14%)	45.8 (8%)		
	Yes	731.03	569.06 (78%)	55.2 (9.7%)	1.23 (0.79, 1.92)	0.356

#### Preoperative Anxiety Sub‐Analysis

Preoperative anxiety assessments with the modified Yale Preoperative Anxiety Scale (mYPAS) [5] were available for 598 surgeries from the VIVID and Magic Coat studies. Among these, 14 surgeries had missing data for the delirium outcome.

When considering only participants with no missing data for the variables listed in Table C.3 and the mY‐PAS score measured during theater sign‐in and induction, the sample size was reduced from 584 to 526 (90%), with 15 participants experiencing emergence delirium. Among these, one child had received Midazolam as premedication (Table C.6).

**TABLE C.6:** Incidence of emergence delirium for patients receiving clonidine/midazolam in the complete case data frame.

	Clonidine		Midazolam	
	No, *N* = 486	Yes, *N* = 40	No, *N* = 468	Yes, *N* = 58
Any emergence delirium	15 (3.1%)	0 (0%)	14 (3.0%)	1 (1.7%)

Due to the low number of patients experiencing emergence delirium and receiving either premedication with Clonidine or Midazolam, a propensity score matched analysis in this subset of data was not feasible.

### Secondary Analysis—Multivariable Logistic Regression

Differences in emergence delirium incidence between the premedication groups were analyzed using a multivariable logistic regression model. The outcome was the occurrence of emergence delirium for each participant (binary yes/no) and the exposure (administration of anesthetic premedication with Clonidine or Midazolam) included as a fixed factor.

Patient demographics and clinical variables were entered as covariates, with interaction terms considered based on exploration. Model selection was performed using stepwise reduction based on the Akaike information criterion (AIC) [6]. The models were then further reduced based on *p* values to include only statistically significant predictors.

Two trials—EEG and LMA and ETT—had no observed cases of emergence delirium and therefore provided no variation in the outcome and do not contribute information to the logistic regression model. Therefore, surgeries from these two trials were excluded to stabilize model estimation and allow the remaining trial effects to be interpreted meaningfully. The sample size was reduced from 4796 to 4575 (95.4%), with 303 surgeries reported as having emergence delirium.

Results of the final fitted model are presented in Table C.7 and Figure C.6. The model included premedication (Clonidine, Midazolam), age, ASA status, sex, surgical specialty, and trial. The included interaction terms were premedication with Clonidine and sex, and premedication with Midazolam and ASA.

**TABLE C.7:** The estimated coefficients and odds ratios (OR) for the logistic model with the outcome of emergence delirium incidence.

Variable	Estimate	OR (95% CI)	*p*	Significance
(Intercept)	−2.466	0.085 (0.055, 0.13)	< 0.001	***
PreMedClonidineYES (ref: NO)	−0.222	0.801 (0.435, 1.473)	0.475	
PreMedMidazYES (ref: NO)	0.613	1.846 (1.195, 2.85)	0.006	**
Age (ref: 0 to 5.9)				
6–8.9	−0.371	0.69 (0.491, 0.969)	0.032	*
9 and over	−0.584	0.557 (0.406, 0.766)	< 0.001	***
ASA (ref: ASA I)				
ASA II	0.186	1.204 (0.861, 1.685)	0.278	
ASA III/IV	−0.111	0.895 (0.534, 1.5)	0.674	
Sex Female (ref: Male)	−0.259	0.772 (0.59, 1.011)	0.06	
Surgery Specialty (ref: General Surgery/Urology)				
Cardiac/Respiratory	−0.261	0.771 (0.174, 3.409)	0.731	
Dental	−0.008	0.992 (0.591, 1.664)	0.975	
ENT	0.714	2.042 (1.306, 3.193)	0.002	**
Gastro	−1.01	0.364 (0.146, 0.907)	0.03	*
Neurosurgery	0.48	1.616 (0.354, 7.375)	0.536	
Oncology	−0.801	0.449 (0.173, 1.164)	0.099	
Ophthalmology	0.374	1.453 (0.444, 4.755)	0.537	
Ortho	0.219	1.244 (0.707, 2.189)	0.448	
Other	0.433	1.542 (0.758, 3.137)	0.232	
Plastics/Burns	0.404	1.497 (0.905, 2.476)	0.116	
Radiology	0.27	1.31 (0.774, 2.217)	0.314	
Rehab medicine	0.414	1.513 (0.592, 3.864)	0.387	
Rheumatology	0.667	1.948 (0.514, 7.376)	0.326	
Trial (ref: POSSUM)				
Deep vs. Awake LMA	−2.9	0.055 (0.013, 0.227)	< 0.001	***
IV vs. INHAL	−3.282	0.038 (0.005, 0.271)	0.001	**
Magic coat	−1.109	0.33 (0.175, 0.62)	0.001	**
MATES	−0.34	0.711 (0.204, 2.48)	0.593	
MIDAZ	1.849	6.355 (3.891, 10.381)	< 0.001	***
Normal values	−3.401	0.033 (0.005, 0.241)	0.001	**
VIVID	−0.689	0.502 (0.216, 1.165)	0.109	
PreMedClonidineYES:Sex Female	0.961	2.613 (1.159, 5.893)	0.021	*
PreMedMidazYES: ASA II	−0.831	0.436 (0.244, 0.777)	0.005	**
PreMedMidazYES: ASA III/IV	0.061	1.063 (0.46, 2.457)	0.886	
Number of observations	4503			

*Note:* Significance: **p* < 0.05, ***p* < 0.01, ****p* < 0.001.

Premedication with Clonidine was associated with a higher odds of emergence delirium in females (OR = 2.09, 95% CI 1.19–3.70, *p* = 0.011) but no significant difference in males (OR = 0.80, 95% CI 0.44–1.47; *p* = 0.475). Midazolam was associated with higher odds of delirium in patients with ASA I (OR = 1.85, 95% CI 1.20–2.85; *p* = 0.006), while there was no significant difference in patients with ASA II (OR = 0.80, 95% CI 0.50–1.31, *p* = 0.378) or ASA III/IV (OR = 1.96, 95% CI 0.93–4.16, *p* = 0.079). Increasing age group was associated with decreasing odds of delirium relative to 0–5.99 years. Relative to general surgery, ENT surgery was associated with increased odds (OR = 2.04, 95% CI 1.31–3.19) and gastroenterology with lower odds (OR = 0.36, 95% CI 0.15–0.91). Although the main effects of sex and ASA were not significant, significant interactions were observed: clonidine × female sex (OR = 2.61, 95% CI 1.16–5.89) and midazolam × ASA II (OR = 0.44, 95% CI 0.24–0.77). Relative to Trial POSSUM which was the trial with the largest sample size, most trials were associated with a lower odds of emergence delirium, though this effect was not significant in MATES and VIVID. MIDAZ was the only trial that was associated with a higher odds of emergence delirium (Figure C.6).
